# Toward predicting photosynthetic efficiency and biomass gain in crop genotypes over a field season

**DOI:** 10.1093/plphys/kiab483

**Published:** 2021-10-18

**Authors:** Beat Keller, Lars Zimmermann, Uwe Rascher, Shizue Matsubara, Angelina Steier, Onno Muller

**Affiliations:** 1 Institute of Bio- and Geosciences, IBG-2: Plant Sciences, Forschungszentrum Jülich GmbH, Jülich 52425, Germany; 2 Field Lab Campus Klein-Altendorf, University of Bonn, Rheinbach 53359, Germany

## Abstract

Photosynthesis acclimates quickly to the fluctuating environment in order to optimize the absorption of sunlight energy, specifically the photosynthetic photon fluence rate (PPFR), to fuel plant growth. The conversion efficiency of intercepted PPFR to photochemical energy (*ɛ_e_*) and to biomass (*ɛ_c_*) are critical parameters to describe plant productivity over time. However, they mask the link of instantaneous photochemical energy uptake under specific conditions, that is, the operating efficiency of photosystem II (*F*_q_′/*F*_m_′), and biomass accumulation. Therefore, the identification of energy- and thus resource-efficient genotypes under changing environmental conditions is impeded. We long-term monitored *F*_q_′/*F*_m_′ at the canopy level for 21 soybean (*Glycine max* (L.) Merr.) and maize (*Zea mays*) genotypes under greenhouse and field conditions using automated chlorophyll fluorescence and spectral scans. *F*_q_′/*F*_m_′ derived under incident sunlight during the entire growing season was modeled based on genotypic interactions with different environmental variables. This allowed us to cumulate the photochemical energy uptake and thus estimate *ɛ_e_* noninvasively. *ɛ_e_* ranged from 48% to 62%, depending on the genotype, and up to 9% of photochemical energy was transduced into biomass in the most efficient C_4_ maize genotype. Most strikingly, *ɛ_e_* correlated with shoot biomass in seven independent experiments under varying conditions with up to *r* = 0.68. Thus, we estimated biomass production by integrating photosynthetic response to environmental stresses over the growing season and identified energy-efficient genotypes. This has great potential to improve crop growth models and to estimate the productivity of breeding lines or whole ecosystems at any time point using autonomous measuring systems.

## Teaser

Cumulative photochemical energy uptake throughout a fluctuating growing season reveals genotypic differences in photosynthetic performance, respiratory losses, and biomass production efficiency.

## Introduction

Photosynthesis is the physiological basis of plant growth and crop yield (Long et al., 2006). Future yield improvement will largely rely on higher net photosynthesis and the transduction efficiency of sunlight into carbohydrates (Zhu et al., 2010; Reynolds et al., 2012). Classical physiology summarizes the energy conversions occurring during plant growth into two major processes (Murchie et al., 2009). First, plant photosynthesis depends on the interception and absorption of the photosynthetic photon fluence rate (PPFR). Second, the intercepted energy must be transduced into biomass. The proportion of PPFR intercepted by the plant relative to the cumulative PPFR, that is, the light interception efficiency (*ɛ_i_*) and the conversion efficiency of intercepted energy into biomass (*ɛ_c_*) describe the potential of biomass accumulation (Zhu et al., 2010). These two coefficients summarize the growth dynamic over time, that is, the chemical conversion of energy into biomass, which in turn allows the physical expansion of leaf area to increase sunlight interception (Evans, 2013). However, important energy losses that occur during the growth process, such as photoprotection and respiration processes, cannot be separated and quantified by *ɛ_c_* or *ɛ_i_*. Additionally, since growth and stress conditions change over the course of the day and the growing season, the actual response of the plant to fluctuating and stress conditions is masked.

Nevertheless, *ɛ_i_* and *ɛ_c_* bare essential information about overall growth performance and are widely used in plant physiology and breeding. It allows to describe biomass production as:

(1) $$\text{Total}\;\text{biomass}=\sum_{\text{Harvest}}^{\text{Sowing}}\text{PPFR}\times \varepsilon_{i}\times \varepsilon_{c}$$

where the PPFR is cumulated over the growing season and multiplied by *ɛ_i_* and *ɛ_c_* (Monteith et al., 1977; Zhu et al., 2010). The ɛ_i_*i*s defined as the ratio of PPFR at the top and bottom of the canopy over a given area. In major crops, *ɛ_i_* was mainly improved by increasing the nitrogen use efficiency and the genetic adaption of growth architecture which resulted in faster canopy closure and higher planting density, respectively (Duvick, 2005; Muurinen and Peltonen-Sainio, 2006). Maize (*Zea mays*) breeding program targeted upright architecture for decades and increased yield successfully (Ford et al., 2008). These efforts were recently enforced by the introduction of genes from the maize ancestor teosinte to further reduce leaf angle (Tian et al., 2019). In soybean (*Glycine max* (L.) Merr.), *ɛ_i_* reaches values >0.9 and is considered highly optimized (Zhu et al., 2010; Koester et al., 2016). The *ɛ_c_* is defined as the efficiency of produced energy biomass relative to the cumulative intercepted PPFR by the canopy over the growing period. In other words, *ɛ_c_* describes the gross photosynthetic efficiency of the full plant stand minus all respiratory losses (Zhu et al., 2010). In contrast to *ɛ_i_*, a general higher *ɛ_c_* through plant breeding has rarely been achieved even when higher photosynthetic rates were observed (Gutiérrez-Rodrı’guez et al., 2000; Murchie et al., 2009; Sinclair et al., 2019). Therefore, *ɛ_c_* was often assumed as constant even though there is natural genotypic variation in crops (Long et al., 2006; Zhu et al., 2010). In the field, *ɛ_c_* is far below the theoretical maximum revealing that photosynthesis is always regulated by environmental fluctuations and stresses (Murchie et al., 2009). Using genetic engineering, a higher *ɛ_c_* was achieved by stimulating electron transport (ET), by improving photorespiration pathway, or by the recovery from photoprotection (Kromdijk et al., 2016; South et al., 2019; López-Calcagno et al., 2020). The *ɛ_c_* may also increase under future elevated CO_2_ levels in the atmosphere which reduce photorespiration (Ainsworth and Long, 2005; Morgan et al., 2005; Terrer et al., 2019). In comparison to *ɛ_i_*, the genetic improvement of *ɛ_c_* was less successful leaving potential for further yield increases by exploiting the genetic variation in that trait (Long et al., 2006; Zhu et al., 2010).

**Figure 1:**
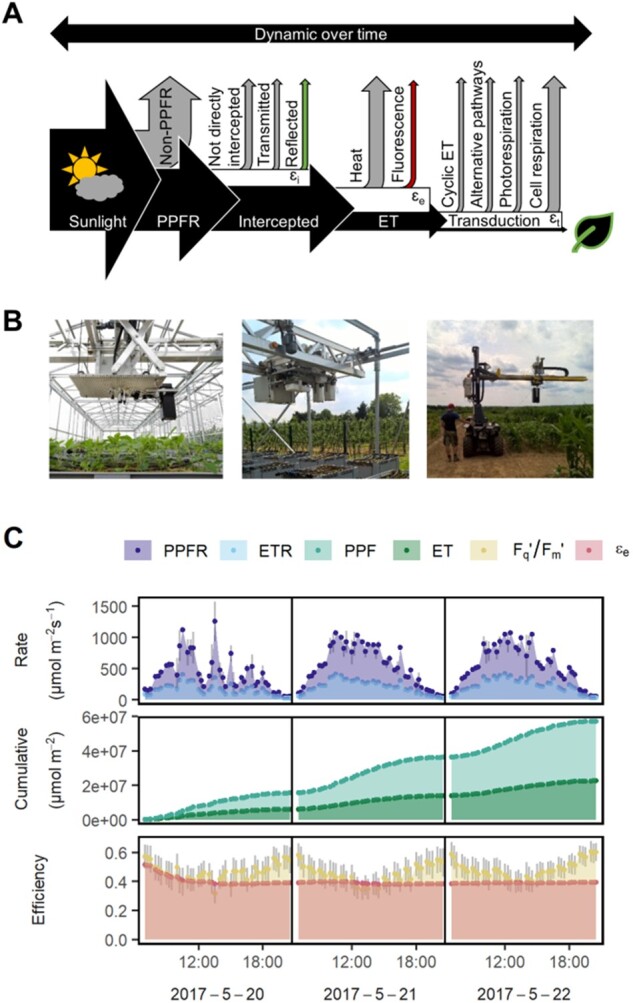
Efficiency of biomass production from sunlight energy under fluctuating conditions was estimated using a high-throughput measuring approach. A, Sunlight energy undergoes several conversions until it is accumulated into biomass. The energy losses occurring under fluctuating conditions are highly dynamic. First, from the full spectrum of sunlight radiation only PPFR can be absorbed by plant pigments. Second, the light interception efficiency (*ɛ_i_*) is depending on the reflected and transmitted light through the plant stand as well as the canopy cover. Hence, the *ɛ_i_* is defined as the ratio of PPFR at the top and bottom of the canopy over a given area. Third, the conversion efficiency of intercepted light energy to photochemical energy (*ɛ_e_*) is derived by measuring ET. The losses through heat and ChlF are depending on the photoprotective acclimation and actual light intensity. The light absorption of non-photosynthetic pigments is negligible. Finally, the conversion efficiency of photochemical energy which is transduced into biomass (ɛ_t_) is depending on the amount of transported electrons used for alternative electron pathways, cyclic ET, photorespiration, and cell respiration including carbohydrate biosynthesis. B, Automated LIFT systems scanned plant canopies inside and outside of the glasshouse as well as in the field in a high spatio-temporal resolution. The dynamic ET under fluctuating conditions was assessed via active ChlF revealing the operating efficiency of photosystem II (*F*_q_′/*F*_m_′). Reflectance was additionally measured using an in-built spectrometer. C, Fluctuating *F*_q_′/*F*_m_′ and PPFR were measured using the LIFT and environmental sensors, respectively. A subset of three subsequent measuring days in soybean is shown. The ETR were derived and cumulative ET and PPF were calculated over these 3 d. The ratio of the cumulative ET and PPF results in the *ɛ_e_* over the growth period. Gray error bars show the standard deviation of the mean per 15 min period for all measurements of the indicated 3 d (*n* = 113–543; total *n* = 36,891 measurements of soybean genotypes).

The genetic variation in *ɛ_c_* is mainly attributed to photosynthetic and respiratory processes, which together determine growth performance. Separating these two processes, the net photosynthesis can be described with the conversion efficiency of intercepted light energy to photochemical energy uptake (*ɛ_e_*). The respiration losses are taken into account via the transduction efficiency of photochemical energy into biomass (ɛ_t_) (Figure  1A). Hence, Equation (1) can be redefined to:

(2) $$\text{Total}\;\text{biomass}=\sum_{\text{Harvest}}^{\text{Sowing}}\text{PPFR}\times \varepsilon_{i}\times \varepsilon_{c}\times \varepsilon_{t}$$

where ɛ_*e*_ is linked to photosynthetic ET. Major losses determining *ɛ_e_* and ɛ_t_ are photoprotective heat dissipation and respiratory processes including carbohydrate biosynthesis, respectively (Zhu et al., 2010; Porcar-Castell et al., 2014).

In order to understand the fundamental relation of energy uptake and biomass accumulation over the growing period (expressed as *ɛ_e_* and ɛ_t_), the dynamic response of photosynthesis to the fluctuating environment needs to be taken into account. Plant photosynthesis acclimates within seconds to fluctuations in light intensity balancing absorbed energy into three different pathways (Butler, 1978; Demmig-Adams et al., 2012; Kono and Terashima, 2014; Lazár, 2015). The first is the photochemical pathway, where the intercepted light energy is converted to ET fueling plant growth (Baker, 2008). In the second pathway, especially in case of excess light, non-photochemical quenching (NPQ) occurs which dissipates intercepted energy as heat (Butler, 1978; Bilger and Bjorkman, 1990). Under field conditions, up to 70% of the absorbed sunlight energy is lost through NPQ decreasing photosynthesis and plant productivity (Endo et al., 2014; Ishida et al., 2014). The third pathway emits intercepted energy as chlorophyll fluorescence (ChlF) which is produced when excited electrons in photosynthetic pigments return to the nonexcited state (Kautsky and Hirsch, 1931; Maxwell and Johnson, 2000). The energy emitted by ChlF is rather minor between 0.5% and 3% (Porcar-Castell et al., 2014). Biomass accumulation is, therefore, highly dependent on the environmental conditions during growth period and the plant’s acclimation to it (Kromdijk et al., 2016; Murchie and Ruban, 2020). In addition, the growing and changing plant canopy causes a complex interplay of light interception and acclimation of photosynthesis (Evans, 2013; Kaiser et al., 2018).

For a detailed view at *ɛ_e_*, the acclimation process of canopy photosynthesis under fluctuating conditions needs to be considered. However, traditional steady-state photosynthesis model as developed by Farquhar et al. (1980) cannot account for environmental fluctuations (von Caemmerer, 2013). These models lay the basis for our mechanical understanding of photosynthesis but need extensions to explain the dynamic processes of photosynthesis and NPQ under field conditions (Nedbal et al., 2007; Murchie and Harbinson, 2014; Rogers et al., 2017). Active ChlF and gas exchange measurements are commonly used to determine photosynthesis in the lab and field using hand-held or bench-top devices (Kalaji et al., 2014). Some devices have been successfully modified to allow long-term monitoring of plant photosynthesis (Song et al., 2016; Hubbart et al., 2018). However, these solutions are not efficient for high-throughput measurements in the field since they are stationary. Therefore, genotype by environment interactions (G × E) of photosynthesis in response to short-time environmental changes and their relation to accumulated biomass over the full growing season are largely unknown (Murchie et al., 2018; Furbank et al., 2019).

Recently, we achieved the estimation of genotype-specific photosynthesis under conditions which are close to field conditions by using a fully autonomous measuring system (Keller et al., 2019a). The key part of the system is a mobile light-induced fluorescence transient (LIFT) device, which enables to induce ChlF from a distance using a fast repetition rate flash (FRRF) (Kolber et al., 1998; Keller et al., 2019b; Osmond et al., 2019). An FRRF generates up to 40,000 µmol photons m^−2^ s^−1^ to induce maximum fluorescence (F_m_′) within 700 µs allowing noninvasive, high-throughput measurements under incident sunlight (Wyber et al., 2018; Keller et al., 2019b). The derived operating efficiency of photosystem II (F_q_′/F_m_′) expresses the proportion of quantum used for ET relative to the absorbed light quantum (Baker, 2008). The LIFT device allows to capture the acclimation of F_q_′/F_m_′ to fluctuating conditions in a high time resolution. Hence, it can be used to calculate ɛ_*e*_ over the growing season. Additionally, the genetic and spatial variation can be observed by acquiring measurements while moving over the plant canopy of different genotypes. The F_q_′/F_m_′ and thereof derived ET rates (ETRs) at given PPFR are linearly related to CO_2_ assimilation in C_4_ plants and in C_3_ plants when photorespiration and cyclic ET is low (Genty et al., 1989; Baker, 2008). In summary, ChlF allows to estimate CO_2_ assimilation in high spatio-temporal resolution capturing acclimation processes in a fluctuating environment. It can serve therefore as fully automated, noninvasive tool to estimate ɛ_*e*_ over the entire growing season in various genotypes.

In this study, we show how to non-destructively estimate plant biomass via measuring the dynamic photochemical energy uptake of different crop genotypes over the growing season. This enabled to assess the fundamental relationship between photosynthetic performance regulated in a short-time (using F_q_′/F_m_′), and the genetic variation of *ɛ_e_* over larger time intervals, up to biomass production. We used autonomous phenotyping platforms operating in the glasshouse and field enabling to capture G × E of photosynthesis on canopy level (Figure  1B). Hence, ETRs are not determined at one time point but integrated over time depending on the dynamic acclimation of photosynthesis under fluctuating conditions (Figure  1C). In that way, we overcome biased photosynthesis measurements by including G × E of actual conditions. We hypothesize that *ɛ_e_* can be used to approximate the accumulated biomass in different C_3_ and C_4_ genotypes. The *ɛ_e_* was estimated based on (1) the genotypic response (slope) of *F*_q_′/*F*_m_′ to increasing or decreasing PPFR (Response_G:PPFR_) while correcting for several selected environmental and spectral variables or (2) ETRs were predicted for every hour of the growing season based on all available variables. Both approaches allow to estimate the absolute amount of electrons transported over the full growing season (seasonal ET) in different soybean and maize genotypes. Furthermore, it allowed to separate respiration from gross photosynthesis estimating *ɛ_t_*. In order to validate and generalize this approach, we used data of seven independent experiments grown under fluctuating conditions in the glasshouse and in the field subjected to various stresses.

## Results

**Figure 2:**
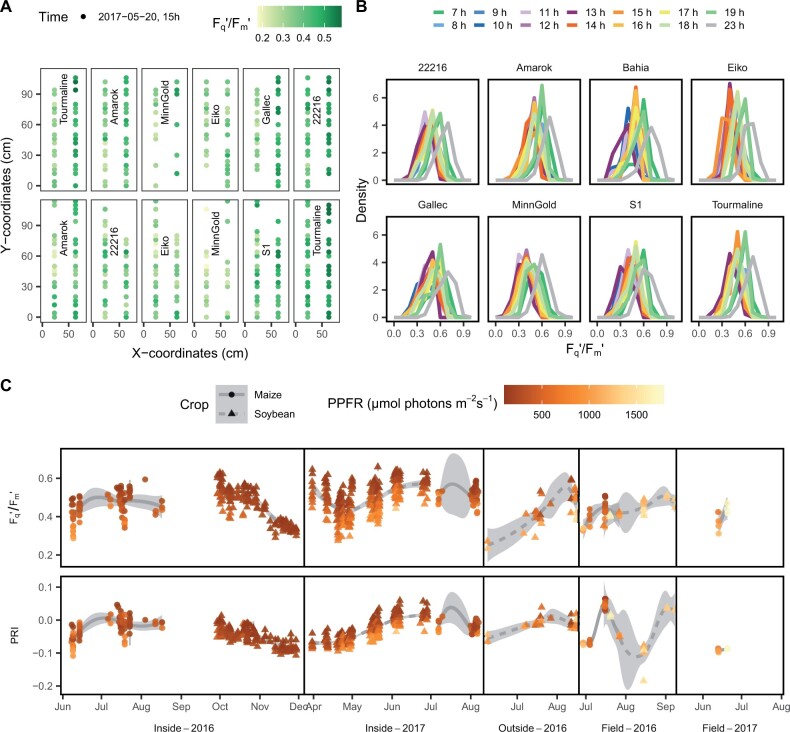
Automated phenotyping systems captured the dynamic of photosynthesis in maize and soybean genotypes in high spatio-temporal resolution. A, The spatial variation for the operating efficiency of photosystem II (*F*_q_′/*F*_m_′) is shown for different soybean genotypes measured on May 20, 2017 at 15 h. Data for the first 12 growth containers are shown. Containers were scanned every hour in two lines using two LIFT devices. B, Distribution of *F*_q_′/*F*_m_′ in the different genotypes per hour of one day (May 20, 2017). C, The *F*_q_′/*F*_m_′ and PRI measured over 2 years in containers inside and outside of the glasshouse and in the field is shown. Gray error bars show the standard error (se) of the mean per hour and crop (*n* = 1–1079, maize: total *n* = 25,014, and soybean: total *n* = 220,881).

Automated measuring scans captured the highly dynamic photosynthetic response in different maize and soybean genotypes grown under fluctuating conditions (Figure  1, A and B). The *F*_q_′/*F*_m_′ showed a clear diurnal pattern (Figure  1C). The variation of *F*_q_′/*F*_m_′ was high in the spatial (over the canopy, Figure  2A) and temporal (per hour, Figure  2B) dimension. Additionally, this data measured over one day gave a first insight into the genetic variation present in the response of *F*_q_′/*F*_m_′ to the fluctuating environment.

**Figure 3:**
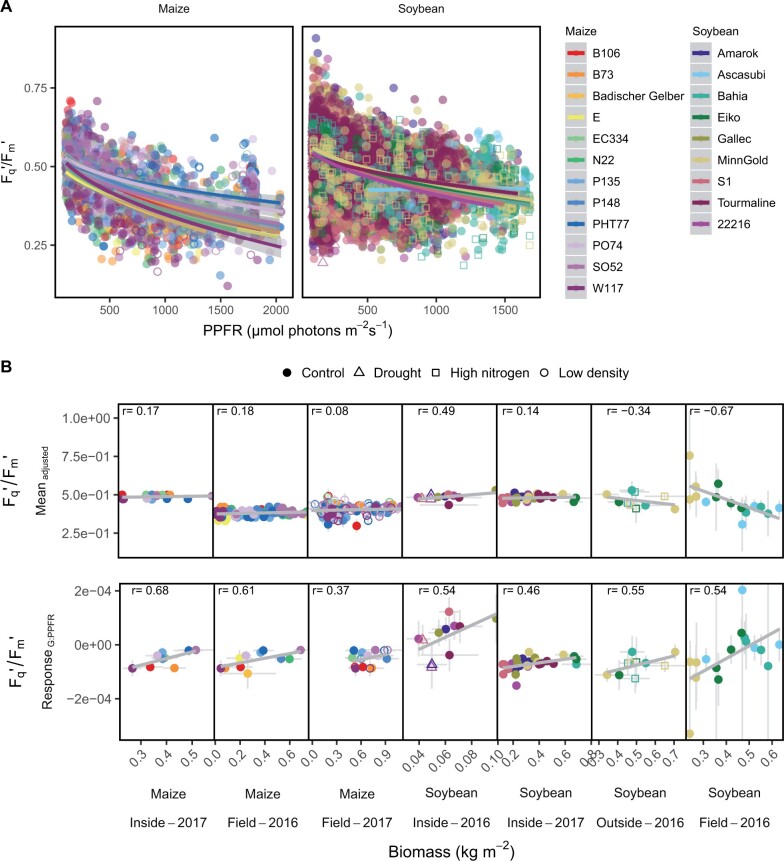
Photosynthetic quantum efficiency (*F*_q_′/*F*_m_′) of soybean and maize genotypes was related to PPFR and biomass. A, The response of adjusted *F*_q_′/*F*_m_′ to PPFR is shown for every genotype. B, The adjusted *F*_q_′/*F*_m_′ means and the genotypic responses of *F*_q_′/*F*_m_′ to PPFR (Response_G:PPFR_) were correlated with measured biomass in all seven experiments. Biomass was shoot biomass, except for the Maize field data it was grain biomass. In soybean, the Response_G:PPFR_ was calculated separately for each container or plot according to Equation (5) using model (3), whereas in maize it was adjusted over all containers and plots using model (4) due to the lower amount of measurements. For the modeling, measurements were previously averaged for every minute, genotype, treatment, and repetition. Gray error bars show the se of the adjusted mean respective response (*n* = 8–504 for *F*_q_′/*F*_m_′; *n* = 1–11 for biomass). Plants were grown under control conditions, except three containers were subjected to drought, five containers were fertilized with high nitrogen and eight maize genotypes were planted in low density in 2017.

In soybean, 206,605 measurements under incident sunlight were taken in 63 d over two seasons from eight genotypes grown in containers inside of the glasshouse (Figure  2C). In containers outside of the glasshouse, 8,304 measurements were acquired over 12 d in three genotypes. Additionally, 5,972 measurements were taken in 8 d in four genotypes over one season in the field. In maize, 12,967 measurements were taken in the glasshouse and 12,047 in the field over two seasons including 31 measuring days and 12 genotypes. This resulted in 16,844 and 3,391 data points in a 1-min resolution for the soybean and maize measuring periods, respectively. The variation of *F*_q_′/*F*_m_′ and the photochemical reflectance index (PRI) over time is shown in relation to PPFR (Figure  2C). The PRI showed a clear seasonal pattern while it was less obvious for *F*_q_′/*F*_m_′. In order to show the Response_G:PPFR_ across all experiments, the *F*_q_′/*F*_m_′ values were first adjusted for the PRI values of the same measurement time point for every genotype and experiment and then correlated with PPFR (Figure  3A). The full models (3) and (4) explained 45% and 53% of the *F*_q_′/*F*_m_′ variance in maize and soybean, respectively. Finally, the adjusted *F*_q_′/*F*_m_′ means and the Response_G:PPFR_ of every genotype in every experiment were extracted and correlated with the accumulated shoot biomass (Figure  3B). Note, that Response_G:PPFR_ is proportional to *ɛ_e_* according to Equation (10). While the adjusted means did not show consistent correlation pattern with biomass, the Response_G:PPFR_ was highly correlated in all experiments. The correlation between Response_G:PPFR_ and biomass was below *r* = 0.46 only in the maize field experiment of 2017. This experiment had the fewest measurements, namely 3,185 originating from only 2 d. The highest correlation of Response_G:PPFR_ and biomass was observed in the maize glasshouse experiment in 2017 with *r* = 0.68 explaining almost 50% of the variation in shoot biomass. The environmental coefficients for PPFR and PRI showed a high importance to determine *F*_q_′/*F*_m_′ in both crops (Supplemental Figure S1). Humidity and the pseudo normalized difference vegetation index (pNDVI) were of lower importance, but relatively higher in maize than in soybean. The interaction with PRI could more than double the prediction accuracy in four experiments and had only small negative effects in two experiments compared to the model results without spectral data (Supplemental Figure S2). Looking at specific time points, *F*_q_′/*F*_m_′ showed also high correlation with biomass toward the end of the growing seasons in the glasshouse (between 0.3 and 0.9 depending on the measurement time) for every hourly measurement run (Supplemental Figure S3). However, this pattern could not be generalized and was not observed in all experiments, especially not in the field and the outside container data. Based on cumulative photochemical energy uptake and cumulative PPFR, the corresponding *ɛ_e_* for every genotype was calculated according to Equations (7)–(9). The *ɛ_e_* ranged between 48% and 62% similarly for soybean and maize genotypes (Table  1). In contrast, the *ɛ_t_* differentiated C_3_ soybean and C_4_ maize genotypes and ranged between 1.2%–5.1% and 5.3%–9.3%, respectively. In summary, *ɛ_e_* was highly correlated with biomass production in all seven experiments.

**Table 1:** The conversion efficiency of intercepted light energy to photochemical energy (*ɛ_e_*), the conversion efficiency of photochemical energy transduced into biomass (ɛ_t_), and the conversion efficiency of intercepted energy into biomass (*ɛ_c_* = *ɛ_e_* ×  ɛ_t_) of 21 maize and soybean genotypes over the growing season

Crop	Genotype	ε_e_	ε_t_	ε_c_
Maize	B106	0.5 (0.001)	0.057 (0.003)	0.029 (0.001)
Maize	B73	0.497 (0.001)	0.065 (0.003)	0.033 (0.002)
Maize	Badischer Gelber	0.479 (0.001)	0.065 (0.005)	0.033 (0.003)
Maize	E	0.53 (0.001)	0.056 (0.005)	0.03 (0.003)
Maize	EC334	0.531 (0.001)	0.053 (0.003)	0.028 (0.001)
Maize	N22	0.527 (0.001)	0.093 (0.005)	0.049 (0.003)
Maize	P135	0.549 (0.001)	0.063 (0.003)	0.034 (0.002)
Maize	P148	0.528 (0.001)	0.075 (0.003)	0.04 (0.001)
Maize	PHT77	0.556 (0.001)	0.07 (0.003)	0.039 (0.001)
Maize	PO74	0.539 (0.001)	0.066 (0.003)	0.035 (0.001)
Maize	SO52	0.557 (0.001)	0.085 (0.003)	0.047 (0.001)
Maize	W117	0.495 (0.001)	0.057 (0.003)	0.029 (0.001)
Soybean	22216	0.499 (0.022)	0.021 (0.004)	0.01 (0.002)
Soybean	Amarok	0.506 (0.023)	0.022 (0.004)	0.011 (0.002)
Soybean	Ascasubi	0.622 (0.029)	0.049 (0.006)	0.03 (0.003)
Soybean	Bahia	0.544 (0.018)	0.051 (0.003)	0.028 (0.002)
Soybean	Eiko	0.533 (0.019)	0.05 (0.004)	0.026 (0.002)
Soybean	Gallec	0.515 (0.023)	0.024 (0.004)	0.012 (0.002)
Soybean	MinnGold	0.503 (0.015)	0.041 (0.003)	0.021 (0.002)
Soybean	S1	0.508 (0.023)	0.012 (0.004)	0.006 (0.002)
Soybean	Tourmaline	0.51 (0.023)	0.036 (0.004)	0.018 (0.002)

The light interception efficiency (*ɛ_i_*) was assumed as constant with a value of 0.9. Adjusted means and se (in brackets) were calculated over all replicates and experiments (*n* = 4–20).

**Figure 4:**
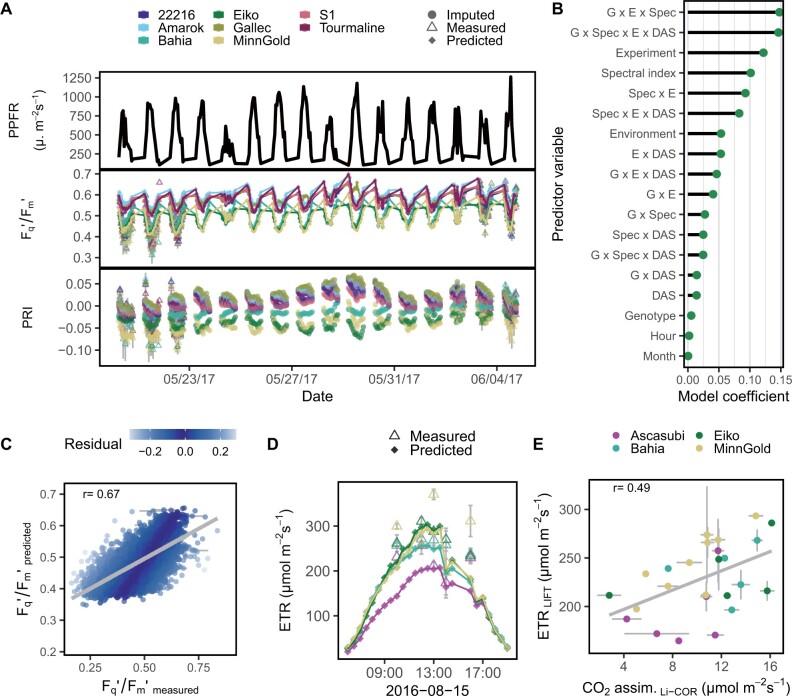
Photosynthetic parameters were predicted over full days based on environmental data and frequently measured photosynthetic quantum efficiency (*F*_q_′/*F*_m_′) values and spectral indices. A, PPFR, *F*_q_′/*F*_m_′ and PRI of soybean genotypes were measured on 83 d over two growing seasons in four experiments. A data subset of 16 d shows the fluctuating growing conditions in the glasshouse. Measured values were averaged per hour and genotype (*n* = 1–64, total *n* = 20,721). Missing spectral data were imputed for the entire growth period in a 1-h resolution. B, Continuous measured environmental data and imputed spectral data were used to model *F*_q_′/*F*_m_′. Model coefficients show the importance of each variable group and their interactions. Interactions were summarized between Genotype (G), environmental variables (E), spectral indices (Spec), and days after sowing (DAS). C, Prediction accuracy for predicted *F*_q_′/*F*_m_′ was evaluated using cross-validation, that is, two-third of the measuring days were used as training set to build model (6) and one-third as validation set to compare predicted versus measured values. The algorithm to solve the linear equation was Ridge Regression. Only predicted values of the validation set are shown. The values were averaged over the different replicates (*n* = 1–8, total *n* = 6,279). The regression line between predicted and measured values is shown in gray. D, Measured and predicted *F*_q_′/*F*_m_′ as well as measured PPFR were used to calculate ETR for every half hour of a day for all four genotypes in the field (*n* = 1–14, total *n* = 239). The colors refer to the genotype legend in E. E, Measured and predicted ETR based on LIFT measurements were compared to CO_2_ assimilation measured by LI-COR in the same half hour and plot (*n* = 1–5 for ETR, total = 89; LI-COR: *n* = 2–161, total *n* = 1,035). Gray error bars show the se of the mean in all panels.

The above results showed that Response_G:PPFR_ was highly correlated with biomass production. However, PPFR is not the only determinant of photosynthesis. A precise estimation of seasonal ET requires ETR in high resolution over the full season to capture the dynamic response to environmental factors. In order to approximate seasonal ET, we predicted missing *F*_q_′/*F*_m_′ for every hour of the growing season. All available data of soybean genotypes, environmental data, and imputed spectral data were used to train the predictive model (6) for *F*_q_′/*F*_m_′ (Supplemental Figure S4). A data subset of 16 d is shown in Figure  4A. Using all available data, the model reached an accuracy of *r* = 0.80 with λ =  0.0055. The relative importance of the model coefficients for *F*_q_′/*F*_m_′ is shown in Figure  4B. The most important interactions were between genotypes, environmental conditions, and spectral variables. Predicted *F*_q_′/*F*_m_′ in soybean reached a cross-validated accuracy of *r* = 0.67 (λ  =  0.0048) using one-third of the measured days as validation set (Figure  4C). As expected, prediction accuracies for *F*_q_′/*F*_m_′ were higher (except for the field data) when spectral data were available in higher time-resolution than ChlF data as compared to the situations in which spectral data was imputed (Supplemental Figure S5). Figure  4D depicts measured and predicted ETR for the soybean genotypes in the field on a day in the season 2016. The predicted ETR showed the usual diurnal pattern in a temporal resolution of 0.5 h. The ETR was correlated (*r* = 0.49) with CO_2_ assimilation measurements taken over the same day in the field (Figure  4E). The predicted ETR for every hour in May and June 2017 is shown in Supplemental Figure S6A. The genotypic interactions with environmental and spectral variables caused the different maxima of the genotypes reached over the days depending on the contemporary environmental conditions. Finally, seasonal ET was calculated according to Equation (8) and correlated with biomass measurements (Supplemental Figure S6B). These correlations did not show a consistent pattern and were all negative. In summary, ETR could be predicted over full seasons with *r* = 0.67 (Figure  4C) but these predictions did not result in accurate biomass prediction (Supplemental Figure S6B). The more simple and robust approach based on Response_G:PPFR_ gave a consistent and reliable predictor (up to *r* = 0.68) for biomass.

## Discussion

In this study, we successfully linked seasonal photosynthetic performance to biomass production in seven experiments under glasshouse up to field conditions. Fully automated LIFT measurements were used to model and predict photosynthesis including G × E, specifically dynamic seasonal ET, in 21 soybean and maize genotypes. We then combined photosynthetic performance and spectral indices to estimate the top of canopy CO_2_ assimilation and biomass production under fluctuating conditions. In previous studies, biomass was estimated based on CO_2_ assimilation under controlled conditions (Dutton et al., 1988) or based on model calculations (Sinclair, 1991). Recently, spectral indices and passive ChlF were used to predict either photosynthesis (Inoue et al., 2008; Camino et al., 2019; Hikosaka and Noda, 2019; Meacham-Hensold et al., 2019) or yield (Swatantran et al., 2011; Montesinos-López et al., 2017; Li et al., 2020; Pique et al., 2020). However, neither passive ChlF nor spectral indices measure directly ETR like active ChlF methods (Schreiber et al., 1986). The relation of photosynthesis and biomass accumulation was studied rarely, for example, in an Arabidopsis mutant compared to the wild-type under lab conditions (Weraduwage et al., 2015) and in wheat spikes under steady-state measurement conditions (Molero and Reynolds, 2020). A general relation of photosynthesis and biomass production under fluctuating conditions was described for the first time in this study.

### Noninvasive estimation of genotypic energy conversion

In the classical physiological approach, *ɛ_c_*, also called radiation use efficiency, is determined destructively via harvesting biomass over time (Reynolds et al., 2000; Koester et al., 2016). In contrast, our approach does not require destructive measurements and is based on noninvasive physiological measurements. It allows the approximation of *ɛ_c_* via *ɛ_e_* using automated *F*_q_′/*F*_m_′ scans for every growing period. This facilitates the identification of genotypes with superior photosynthetic performance under specific conditions. Indeed, there was a high genetic variation for *ɛ_e_* and ɛ_t_ in the soybean and maize genotypes (Table  1). The *ɛ_e_* values of around 0.5–0.6 indicate high losses through NPQ processes under fluctuating conditions. Similar NPQ losses were reported in a field study in rice (Ishida et al., 2014). The recovery from photoprotective NPQ state was recently targeted in order to develop more efficient plants (Long et al., 2006; Kromdijk et al., 2016). These plants were genetically engineered, leaving the field open for further screening of the natural genetic variation in that trait (Murchie and Ruban, 2020). Whereas the *ɛ_e_* values were comparable in C_3_ soybean and C_4_ maize genotypes, the *ɛ_t_* values reached 3.4% and 6.7% on average, respectively. These significantly lower ɛ_t_ values in the C_3_ crop are partly explained due to the photorespiration which does not occur in C_4_ plants (Zhu et al., 2010). The *ɛ_c_* of soybean and maize genotypes reached up to 3.0% and 4.9%, respectively, a lower efficiency than previously reported for C_3_ (5.1% for wheat and barley) and C_4_ (7.4% for maize) crops (Amthor, 2007). This was probably due to suboptimal growth conditions and less productive genotypes, for example, the two soybean genotypes with *ɛ_c_* below 1.1% were cold sensitive and not adapted to grow in colder climate (Keller et al., 2019a). In summary, the presented approach has a great potential in physiological breeding, for example, as an early selection trait to identify resource-efficient genotypes with low NPQ losses. Additionally, crop growth models can be augmented with genotype-specific *ɛ_c_* and ɛ_t_ values.

### Environmental conditions and canopy structure influence photosynthetic efficiency

The accuracy of estimated *F*_q_′/*F*_m_′ values and, therefore, of seasonal ET calculations, depends on the model’s ability to capture relevant environmental interactions. Based on our previous investigation, we focused on the environmental interactions of *F*_q_′/*F*_m_′ with PPFR, humidity, and spectral indices (Keller et al., 2019a). The influence of PPFR to *F*_q_′/*F*_m_′ is connected to NPQ mechanism within the light-harvesting complex of photosystem II which dissipates excess energy as heat (Baker, 2008; Ruban et al., 2012). On leaf level, PRI is linked to changes in NPQ and light use efficiency (Gamon et al., 1992; Barton and North, 2001) and varies within the canopy (Foo et al., 2020). However, on canopy level, it became clear that the PRI is very sensitive to changes in canopy structure and chlorophyll content (Garbulsky et al., 2011). Structure-related changes are likely to dominate the information contained in PRI, especially during seasonal measurements (Gitelson et al., 2017). Additionally, PRI was strongly linked to leaf area index probably further connected to light distribution in the canopy (Wu et al., 2015). In agreement, we found a clear visible seasonal pattern of PRI. These multiple factors represented by PRI probably explain the high importance of PRI interaction with *F*_q_′/*F*_m_′ in the models (3) and (4) (Supplemental Figure S1). In addition to PRI and PPFR, *F*_q_′/*F*_m_′ was adjusted for minor effects of pNDVI and humidity. The effect of humidity on photosynthesis was mainly studied using vapor pressure deficit, which is positively correlated with leaf transpiration rate and negatively with CO_2_ assimilation rate (Morison and Gifford, 1983; Peterson, 1990; Lawson et al., 2002; Ribeiro et al., 2004; Zhang et al., 2017). The NDVI correlated with plant productivity although saturating at high leaf area index (Gamon et al., 1992; Ji and Peters, 2003). The pNDVI was associated with canopy structure, based on differences of measurements on fixed, flat leaves and leaves with natural leaf angles (Keller et al., 2019a). That structure indeed may define photosynthetic performance was recently demonstrated by linking the wheat spike photosynthesis to biomass production (Molero and Reynolds, 2020). The PRI and pNDVI bare information about canopy structure and leaf area index which potentially accounts for within canopy photosynthesis. In summary, the inclusion of spectral indices into the *F*_q_′/*F*_m_′ models substantially improved biomass estimation (Figure  3B compared to Supplemental Figure S2), partly accounting for differences in light distribution within the canopy and leaf area index.

### Photosynthetic response to light intensity corresponds to biomass

The dynamic response of *F*_q_′/*F*_m_′ to environmental changes and stresses complicates the estimation of seasonal photosynthetic performance. Adjusted means of *F*_q_′/*F*_m_′ over the growing season showed correlation with biomass but not in a consistent manner (Figure  3B;Supplemental Figure S3). This could have two explanations. First, the *F*_q_′/*F*_m_′ measurements are biased toward specific PPFR conditions, which are not critical for biomass accumulation, for example, by the overrepresentation of low light conditions in the measured periods. Second, proximity sensed *F*_q_′/*F*_m_′ tends to change with plant height when the target leaves are not in the focus of the excitation flash, as was shown in our previous study using the LIFT device (Keller et al., 2019a). Therefore, the genotypic interactions with environmental and spectral variables were additionally calculated as unbiased estimates of photosynthetic performance toward both influences. Indeed, the Response_G:PPFR_ was highly correlated to biomass in all seven experiments (*r* ranged from 0.37 to 0.68; Figure  3B). Hence, the Response_G:PPFR_ explained up to 46% of the variation for biomass. The seven experiments represented a wide range of genotypes and environments including glasshouse and field conditions as well as (stress) treatments such as drought, cold, and low chlorophyll content. We conclude that the photosynthetic Response_G:PPFR_ accounts for different environmental conditions or stresses and is tightly linked to biomass accumulation.

### Predicted photosynthesis over whole seasons

For days which had no measurement data, ETR could be predicted based on environmental variables only (Figure  4C). The imputation of missing PRI data on days with no measurements decreased the prediction accuracy (Supplemental Figure S5). Hence, spectral reflectance or sun-induced ChlF parameters derived from airplanes or satellites could improve *F*_q_′/*F*_m_′ predictions and extend the predictions to wider areas (Drusch et al., 2017; Mohammed et al., 2019). The measured and predicted ETR allowed to estimate CO_2_ assimilation in soybean genotypes with *r* = 0.49 (Figure  4E). The ETR does not account for photorespiration and other active electron sinks downstream of photosystem II (Baker, 2008). Therefore, low prediction accuracy might be expected. However, comparing leaf measurements directly, the relation of LIFT-derived to gas exchange measurements was shown before with high accuracy (*R*^2^ = 0.94) (Pieruschka et al., 2010). In this study, the comparison of gas exchange measurements on leaf level with the predicted ETR on canopy level might explain the lower prediction accuracy. Furthermore, the estimation of seasonal ET was probably biased because it was not independent from plant height, canopy structure, and interactions with further environmental variables (Supplemental Figure S6). In consequence, the approach to model *F*_q_′/*F*_m_′ using Response_G:PPFR_ was more robust and tighter linked to biomass production.

### Efficiency estimates: caveats and challenges

Improvements in model accuracy and efficiency estimates need to be considered in four areas: First, the root biomass, canopy cover, and *ɛ_i_* can be measured additionally to replace the corresponding model assumptions (Sinclair and Muchow, 1999). The time resolution of spectral measurements could be improved, for example, by acquiring frequent airborne measurements. Second, the focus of the LIFT flash requires a higher dynamic range to ensure unbiased measurements at changing measuring distances (Keller et al., 2019a). Third, the relation between top of canopy photosynthesis and within canopy photosynthesis, that is, the heterogeneities of microclimatic conditions within the canopy, needs to be addressed in more detail (Schurr et al., 2006; Nichol et al., 2012; Zhu et al., 2012). The spectral indices accounted partially for the top of canopy structure in our simplified one-layer canopy model. However, the inner, light-limited canopy contributed almost 50% to the total canopy photosynthesis based on 3D canopy reconstruction in rice (Song et al., 2013). Such 3D canopy models could improve the estimation of *ɛ_e_* for the whole canopy associating every point in the canopy with its predicted light intensity and specific photosynthetic response. Around 70% of the intercepted PPFR is absorbed by the outer canopy, which consequently dissipates most of the excess energy through NPQ (Song et al., 2013). Therefore, the derived *ɛ_e_* in our study based on top of canopy measurements are relevant, but rather underestimated because the remaining 30% of the absorbed energy was likely used at higher *F*_q_′/*F*_m_′ in the inner canopy. In turn, *ɛ_t_* could not be calculated precisely and was rather overestimated. Fourth, regarding temperature, a major effect of temperature was found not for *F*_q_′/*F*_m_′ but for ET efficiency (Keller et al., 2019a). ET efficiency and its response to temperature may have potential for further improvement of biomass prediction, especially in the light-limited, inner canopy, since positive correlations with biomass were observed in all experiments (Supplemental Figure S7). In conclusion, further studies are necessary to improve biomass prediction based on seasonal ET of the full canopy. In this study, we demonstrated that top of canopy *F*_q_′/*F*_m_′ measurements were sufficient to screen for NPQ efficient genotypes with high *ɛ_e_* and showed that Response_G:PPFR_ highly correlated with shoot biomass production.

We presented a crop growth model approach which uses the fundamental response of photosynthesis to environmental (stress) factors to predict biomass in various soybean and maize genotypes grown under different conditions. This noninvasive and automated approach can be refined to estimate biomass production from individual plants (or breeding lines) up to global ecosystems under any environmental conditions. Additionally, it could lead to a global map of photosynthesis and a better understanding of limiting environmental factors based on global measurements as in the oceans (Falkowski et al., 2017) and may be combined with gross plant production models based on satellite data (Drusch et al., 2017; Pique et al., 2020).

## Materials and methods

The contribution of photosynthesis on biomass accumulation was assessed for 21 genotypes. The *ɛ_e_* and ɛ_t_ were estimated in seven independent experimental data sets under natural fluctuating conditions in the glasshouse up to field conditions.

### Plant material

In total 12 maize (*Z.* *mays*) and 9 soybean (*G. max* (L.) Merr.) genotypes were evaluated. Maize genotypes were selected within the German plant phenotyping network and provided by the Leibniz Institute of Plant Genetics and Crop Plant Research. These genotypes represent a diverse set with contrasting shoot and root traits. Soybean genotypes differ in cold tolerance and include the chlorophyll-deficient mutant MinnGold (Campbell et al., 2015). The cultivars Ascasubi, Gallec, and Tourmaline are registered in the European common catalog of varieties (European Commission, 2016). The remaining five soybean genotypes are described by Keller et al. (2019a) originating mainly from the Agroscope breeding program in Changins, Switzerland. Maize and Soybean genotypes evaluated in each experiment are listed in Supplemental Table S1.

### Growth conditions

In total seven experiments took place at Campus Klein Altendorf (University of Bonn, Germany, 50°37′ N, 6°59′ E) in 2016 and 2017. The plants were grown under incident sunlight and fluctuating conditions in the glasshouse up to the field. Three experiments were carried out in containers inside the glasshouse and one experiment in containers outside of the glasshouse. The facility is an unheated glasshouse without artificial lightning (called Mini-plot) as described by Thomas et al. (2018). An automated positioning system allows measurement scans over plant canopies growing in containers inside and outside of the glasshouse (Figure  1B). Other three experiments were conducted directly in the field. The sowing and harvest date of all experiments are shown in Supplemental Table S1. The field site has a loamy-clay silt soil (luvisol) and containers were filled with soil from there (Hecht et al., 2016).

#### Containers

Plants were grown in containers (111 × 71 × 69 cm, AUER Packaging, Belgium) with a volume of 535 L under natural fluctuating sunlight inside and outside of the glasshouse. Control plots were watered using drop irrigation and fertilized after common agriculture practice. All containers were weeded manually. Soybean and maize genotypes were sown in two rows per container (40-cm row distance) in a density of 30 and 20 plants per square meter, respectively.

Inside the glasshouse, Maize and soybean genotypes were grown under controlled conditions as described in Keller et al. (2019a) and, a subset of genotypes, under drought conditions. Three genotypes (Amarok, S1, and 22216) received about 70% of water supply in October and 90% in November 2016 compared to control conditions. Genotypes were replicated in one to four containers per treatment (see Supplemental Table S1).

Outside of the glasshouse, three soybean genotypes (MinnGold, Eiko, and Bahia) were sown in 2016. Containers were watered, with the same irrigation system as inside, once or twice a week depending on the amount of rain. Genotypes were grown under controlled (no application of fertilizer) and high nitrogen conditions (8 g of nitrogen in the form of ammonium nitrate dissolved in water was applied on 14 July 2016). MinnGold and Bahia genotypes were replicated in two containers per treatment and Eiko in one.

#### Field

Soybean and maize genotypes grown in the field were not irrigated. Fertilizer and plant protection agents were applied after good agricultural practice as described in Keller (2018). For the maize genotypes, the plot size was 3 × 3 m with a sowing density of 10 seeds m^−2^ as control and 5 seeds m^−2^ for low-density treatment. Maize genotypes were sown in 7–11 repetitions in 2016 and 2017 in a randomized block design (Supplemental Table S1). For the soybean genotypes, the plot size was 4 × 1.5 m with a sowing density of 100 seeds m^−2^ due to low germination rates. Four genotypes (Ascasubi, MinnGold, Eiko, and Bahia) were sown in four repetitions in 2016.

### ChlF and spectral measurements

The LIFT-REM (Soliense Inc., New York, NY, USA) device was used to measure photosynthesis by probing ChlF from the distance (Kolber et al., 1998; Keller et al., 2019b). The LIFT device operated in a high-throughput mode scanning the top of the plant canopy (Figure  2A). The scans were performed in constant speed (about 10 cm s^−1^) using FRRFs in a 2 s interval. All measurements were acquired under incident sunlight. The measurement direction was toward the south to avoid shading of the target leaves.

The FRRF generates an excitation power of about 40,000 µmol photons m^−2^ s^−1^ at 60 cm distance using 300 excitation flashlets in 2.5-µs interval. The ChlF yield after the 1st and 300th excitation flashlet equals minimal fluorescence and maximal fluorescence (*F*_m_ in the dark, *F*_m_′ in the light), respectively (Keller et al., 2019b). The difference between both ChlF yields results in the variable fluorescence (*F*_v_ in the dark, *F*_q_′ in the light) used to calculate *F*_q_′ /F_m_′. The measured area in the focus of the LIFT instrument at 60 cm distance was a circle of about 7 cm^2^. The light spectrum on the measurement area was recorded by the built-in STS-VIS spectrometer (Ocean Insight, Orlando, FL, USA) from 400 to 800 nm with a resolution of 0.46 nm through the LIFT lens. Spectral measurements with 200 -ms integration time were acquired in-between the FRRFs.

Inside and outside of the glasshouse, crop canopies of different genotypes growing in containers were scanned in 3 × 30 cm steps. This resulted in about 18 and 36 measurements per container when using one and two LIFTs, respectively. The measuring distance was between 1.4 and 0.8 m depending on the plant height. The scanning of all containers was repeated every hour up to 5 d in a week as described by Keller et al. (2019a). In the containers outside of the glasshouse, the measurements were done sporadically over the growing season. All measurements were acquired in fully automated measurement runs. Regarding the control containers inside the glasshouse, the LIFT data of soybean and maize described by Keller et al. (2019a) were used.

Field measurements were taken by an autonomous field robot (*FieldCop*) or by a self-built, manually driven field cycle (*field4cycle*). The *field4cycle* had a track width of 3 m and measured on top of the plot within the track. The autonomous field robot (Raussendorf GmBH, Obergurig, Germany) was equipped with a flexible boom (Lüttich Ingenieure GmbH, Dohna OT Borthen, Germany) allowing measurements from up to 4 m in height and 3.8 m next to the machine track. The field robot took measurements in soybean on August 15, 2016. All other days in the field were measured with the field4cycle. About 15–20 measurements were acquired per plot. The distance between canopy and LIFT lens was kept between 50 and 80 cm. Measurement runs over the full field were done sporadically over the growing season.

### Gas exchange measurements

Gas exchange measurements were carried out in the field on August 15, 2016 using two LI-6400XT devices (LI-COR, Inc., Lincoln, NE, USA) with transparent chamber heads. A fully expanded leaf was measured in horizontal position on top of canopy. The transparent chamber head allowed ambient sunlight to drive photosynthesis. Plots were measured alternately for around 15 min using two LI-CORs logging data every 10 s. Measurements with a stability factor of less than 0.6 were filtered out. Air temperature in the chamber was controlled to match the ambient temperature in the field. The air was coming from inside a 50 L canister with open cap to ensure stable CO_2_ content. The LI-CORs were matched every 45–60 min.

### Harvest and biomass

Plants were harvested when most genotypes in a trial reached full maturity. Regarding the experiments carried out in containers, 2–7 plants per container were harvested manually. Plant material was dried for 48 h at 70°C and individual plants were weighed. Maize field plots were harvested by a maize harvester and fresh weight of grain biomass was weighted for every plot. The dry weight per plot was calculated based on the water content of the biomass. The water content was measured, from a subsample of freshly shredded and well-mixed harvested biomass from each plot, as the ratio of 200 g biomass before and after drying. From the soybean field trial, a plot area of ∼3 m^2^ was harvested and weighed after drying for 24 h at 100°C. This dry weight was corrected for the number of harvested plants per plot. The harvest date was the same for each experiment except for the soybean field trail where genotypes were harvested on different dates (Supplemental Table S1).

### Environmental data

Temperature, humidity, and PPFR data were recorded at ∼1.5-m above ground every minute. Up to three stations recorded data inside of the glasshouse, one outside at the containers, and further three in the field. The sensor system was described by Keller et al. (2019a). Environmental records were averaged per minute for each condition (inside the glasshouse, outside the glasshouse, and field) and associated with every LIFT measurement performed in the same minute and condition.

### Data processing

ChlF data were processed as described earlier using ChlF induction and relaxation (Keller et al., 2019a, 2019b). Spectral values of every measurement were binned and averaged to even numbers of wavelengths. Reflectance was calculated using a gray reference look up table as described by Keller et al. (2019a). Briefly, every spectral measurement of a plant was divided by a spectrum taken on a gray reference. Since such a reference was not immediately available, spectra were corrected with a reference spectrum on a look up table at similar light intensity. The look up table data were generated between May 15 and May 18, 2017 within the diurnal measurements. In contrast to measurements inside the glasshouse, in the field not many reference measurements were taken therefore the look up table was created by scaling the spectra relative to the PPFR when the measurement took place. In that way, spectra were generated from 200 to 1,500 PPFR. The following variables were derived based on spectral data. Absorbance was calculated between the absorbance maxima of the chlorophyll, at 420–500 and 640–690 nm:

$$\text{Absorption}=(\sum_{500\;\text{nm}}^{420\;\text{nm}}\text{Spectralsignal}+\sum_{690\;\text{nm}}^{640\;\text{nm}}\text{Spectralsignal})/\sum_{800\;\text{nm}}^{400\;\text{nm}}\text{Reference}\;\text{signal}$$

Three established spectral indices, PRI, normalized phaeophytinization index (NPQI) and NDVI, were calculated using the following wavelengths:

PRI =  (R530 − R570)/(R530 + R570) adapted from Gamon et al. (1992)

NPQI =  (R416 − R436)/(R416 + R436) adapted from Peñuelas and Filella (1998)

NDVI =  (R750 − R706)/(R750 + R706) adapted from Frampton et al. (2013) shifting the selected red and near-infrared wavelength toward the end and the beginning of their spectral range, respectively.

Spectral indices were calculated based on the corrected reflectance spectrum or directly on the raw digital numbers of the spectrometer output. In the latter case, uncorrected indices were denoted with a “p”, for example, pNDVI. Pseudo indices were used additionally since the spectra used for correction are only an approximated white reference spectrum based on the described look up table. The irradiation variable recorded the signal at 680 nm detected by the LIFT before the FRRF. For the following analysis only light-adapted measurements were selected (PPFR > 100 µmol photons m^−2^ s^−1^). Data points more distant than 2.5 times the interquartile range from the first respective third quantile were removed for every spectral variable per crop and treatment, for every environmental variable per month, and for biomass per experiment. *F*_q_′/*F*_m_′ and spectral values were averaged per minute, repetition, treatment, and genotype.

### Modeling on observed data

Based on the *F*_q_′/*F*_m_′, spectral values and its associated environmental variables derived over the measuring periods in a 1-min resolution, adjusted *F*_q_′/*F*_m_′ means and Response_G:PPFR_ were calculated for every genotype. In maize, *F*_q_′/*F*_m_′ values (*y_ijklm_*) can be described under different environmental conditions including genotypic interaction with environmental covariates using the following linear model:

(3) $$y_{\text{ijklm}}=µ+E_{i}+G_{j}+R_{k(il)}+H_{m}+P_{m}+I_{m}+N_{m}+{\;\text{PI}}_{m}{+\text{GP}}_{\text{jm}}+{\text{GI}}_{\mathrm{jm}}+{\text{GPI}}_{\mathrm{jm}}+\varepsilon_{\text{ijklm}}$$

where *μ* is the intercept, *E_i_* is the fixed effect for the experiment *i*, *G_j_* is a fixed effect for the genotype *j*, *R_k(il)_* is a fixed effect for the replicate *k* nestled within experiment *i* and treatment *l*, *H_m_* is a fixed effect for the humidity value at the time point *m* of the measuring period in the 1-min resolution, *P_m_* is a fixed effect for the PPFR value at the time point *m*, *I_m_* is a fixed effect for the PRI value at the time point *m*, *N_m_* is a fixed effect for the pNDVI value at the time point *m*, PI_*m*_ is the interaction between the PPFR and PRI value at time point *m*, GP_*jm*_ is the interaction between genotype *j* and PPFR value at time point *m*, GI_*jm*_ is the interaction between genotype *j* and the PRI value at the time point *m*, GPI_*jm*_ is the interaction between genotype *j*, PPFR, and PRI value at the time point *m*, and *ε_ijklm_* is the error term.

In soybean, genotypic interactions were fitted on plot level in order to account for spatial effects between the plots or containers using the following linear model:

(4) $$y_{\text{ijklm}}=µ+E_{i}+G_{j}+R_{k(il)}+H_{m}+P_{m}+I_{m}+N_{m}+{\;\text{PI}}_{m}+{\;\text{GR}}_{jk(il)}+{\;\text{GPR}}_{\text{jkm}(\text{il})}+{\;\text{GIR}}_{\text{jkm}(\text{il})}+{\;\text{GPIR}}_{\text{jkm}(\text{il})}+\varepsilon_{\text{ijklm}}$$

where G_*j*_, *GP_jm_*, *GI_jm_*, and *GPI_jm_* were fitted with an interaction of every replicate *k* nestled within each experiment *i* and treatment *l*. This results in the interaction terms *GR_jk(il)_*, *GPR_jkm(il)_*, *GIR_jkm(il)_*_,_ and *GPIR_jkm(il)_* which allow the fitting of *F*_q_′/*F*_m_′ values on plot level. These interaction terms were omitted in maize because less data per plot was available. The *H_m_*, *P_m_*, *I_m_*, and *N_m_* are effects of covariates which were chosen for modeling based on previous analysis of important factors determining *F*_q_′/*F*_m_′ (Keller et al., 2019a). Note that the covariates have a numeric value at every time point *m* which multiplied by the regression coefficient (β) results in the effect at time point *m*, for example, GP = {*GP_jm_*} = β_GP_Z_G_X_P_, where β_GP_ is a vector with a regression coefficient for every genotype introduced as Response_G:PPFR_, Z_G_ is a design matrix for the genotypes, and X_P_ is a vector of PPFR values. The β_GP_ and β_GPR_ coefficients of GP for maize and GPR for soybean, respectively, were extracted using the emtrends command of the emmeans R package (Lenth, 2019). These interaction coefficients, β_GP_, and β_GPR_, were calculated as followed:

(5) $${\text{Response}}_{\text{G:PPFR}}=\partial \;E\;\left(y\right)/\partial \;X_{P}$$

where ∂*E* (*y*) denotes the delta of the expected (fitted) *F*_q_′/*F*_m_′ values and ∂*X*_P_ the corresponding delta of the PPFR values. Hence, the Response_G:PPFR_ expresses the slope of *F*_q_′/*F*_m_′ with increasing or decreasing PPFR for every genotype (Eeuwijk et al., 2016). It is also called the genotypic interaction of *F*_q_′/*F*_m_′ with PPFR. Finally, the adjusted mean of *F*_q_′/*F*_m_′ and Response_G:PPFR_ were correlated with measured biomass and the Pearson correlation coefficient (*r*) was calculated.

### Predictive modeling for time points without measuring data

All described environmental and spectral variables were used for predictive modeling. In addition, descriptive variables for hour, month, and days after sowing (DAS) were included. For the spectral variables, missing values (when no measurement data was available) were imputed separately for every genotype for every hour of the growing season with averaged PPFR values >100 µmol photons m^−2^ s^−1^. This was done based on the first six principal components derived from the available numeric environmental and descriptive variables, that is, hour, DAS, humidity, temperature, PPFR, the square root of PPFR, irradiance, and the calculated spectral variables. The regularized iterative principal components analysis algorithm implemented in the missMDA R package was used (Josse and Husson, 2016).

The *F*_q_′/*F*_m_′ values (*y_ijklm_*) were predicted using Ridge Regression implemented in the glmnet R package (Friedman et al., 2010). The following random-effect model adapted from Jarquín et al. (2014) was used:

(6) $$y_{\text{ijklm}}=µ+E_{i}+G_{j}+v_{m}+w_{m}+Gw_{\text{jm}}+\varepsilon_{\text{ijklm}}$$

where *μ* is the intercept, *E_i_*∼*N*(0,σ^2^_E_) is a random effect for the experiment *i*, *G_j_*∼*N*(0,σ^2^_G_) is a random effect for the genotype *j*, v = {*v_m_*} is a matrix with numeric columns for the month to account for seasonal trends, for the hour of the measurement to account for daily trends, for the irradiation, the absorbance as well as the reflectance and rows for every time point *m* of the measuring period in the 1-h resolution. It was assumed that v∼N(0,Vσ^2^_v_), where V is the covariance matrix of *v* (i.e. V = vv′). Additionally, w =  {*w_m_*} is a matrix with columns for PRI, pNDVI, NPQI, DAS, humidity, temperature, PPFR as well as the square root of PPFR and rows for every time point *m* with w∼N(0,Ωσ^2^_w_), where Ω is the covariance matrix of w (i.e. Ω = ww′). The term Gw∼N(0,Z_G_Z′_G_ ∘ Ωσ^2^_Gw_) is the interaction between every genotype j and the environmental values in w at time point *m*, where Z_G_ is the design matrix for the genotypic effects and ∘ denotes the Hadamard product. Lastly, *ε_ijklm_*∼*N*(0,σ^2^_ε_) is the error term. The vector of all predictor coefficients, β, is restricted by λ which is determined by internal cross-validation (Friedman et al., 2010). The ridge parameter λ shrinks the coefficients of correlated variables according to the *L*_2_ norm to reduce their variance (Hastie et al., 2009). Datapoints with associated PPFR values >100 and <2,000 µmol photons m^−2^ s^−1^ were averaged per hour and plot for the modeling. All numeric covariates were standardized with mean = 0 and standard deviation = 1. The *F*_q_′/*F*_m_′ values were predicted for every hour of the growing season.

#### Cross-validation of predicted *F*_q_′/*F*_m_′ values

Cross-validation was done by using the data of two-third of the measuring days as training set and the remaining measuring days as validation dataset. Then, *F*_q_′/*F*_m_′ values were predicted for the days of the validation set. Pearson correlation coefficient (*r*) was used to assess prediction accuracy of measured and predicted values. This procedure was repeated once with available spectral data in the validation set and once without. The first case assumes that additional spectral data is available from other sensor, for example, mounted to unmanned aerial vehicles.

### Calculations of conversion efficiencies

In-between light interception and biomass production, light absorption drives ET while excess energy is dissipated as heat. In order to separate photochemical energy uptake from heat losses, seasonal ET and *ɛ_e_* were calculated. The respiratory losses during biomass accumulation are considered by the calculation of ɛ_t_.

#### Seasonal ET

First, ETR were calculated for every genotype in an hourly resolution over the growing season:

(7) $$\text{ETR}=F_{q}{}^{\prime }/F_{m}{{}^{\prime }}_{\text{predicted}}\times \text{PPFR}\times 0.5\times \varepsilon_{i}$$

whereas the factor of 0.5 approximates the fraction of PPFR which is received by photosystem II (Baker, 2008). The *ɛ_i_* was assumed to be 0.9 over the full season because no precise measuring data was available. Indeed, the radiation interception efficiency can reach values up to 0.9 in soybean stands (Koester et al., 2016). The intercepted light energy was assumed to fuel 100% photochemistry because the contribution of nonphotosynthetic pigments to light absorption is minor (Porcar-Castell et al., 2014). Hourly ETR values were estimated based on F_q_′/F_m_′ values derived from models (3) and (4) using simple extrapolation based on Equation (5); and from model (6) including multiple environmental interaction effects for every hour. For the simple extrapolation, the Response_G:PPFR_ and a common intercept equal to the average of the *F*_q_′/*F*_m_′ in low light (PPFR < 105 µmol photons m^−2^ s^−1^) over the entire season per crop were used to calculate hourly *F*_q_′/*F*_m_′ values. The complexity of light scattering and photosynthesis within the canopy could not be addressed in this study. The canopy photosynthesis was simplified and assumed to origin from one heterogenous layer. In order to summarize photochemical energy uptake over the entire growing season,

(8) $$\text{Seasonal}\;\text{ET}=\sum_{\text{Senescence}}^{\text{Germination}}\left(\;\text{ETR}\times 3600\;s\right)$$

was calculated in µmol electrons m^−2^ for every genotype (maize) or even every plot (soybean) in every experiment based on the ETR values calculated for every hour. The growing season was defined as starting from germination 21 DAS and ending at senescence 21 d before harvest. PPFR values were hourly averaged.

#### Conversion efficiency of intercepted light energy to photochemical energy uptake

The *ɛ_e_* can be calculated from seasonal ET, combining Equations (7) and (8), relative to the intercepted light energy, which results in:

(9) $$\varepsilon_{e}=\sum_{\text{Senescence}}^{\text{Germination}}\left(F\;\mathrm{q'/F}\;m\;\prime \text{predicted}\times \text{PPFR}\right)/\sum_{\text{Senescence}}^{\text{Germination}}\text{PPFR}$$

where the term $\sum_{\mathrm{Senescence}}^{\mathrm{Germination}}$ (*F*_q_′/*F*_m_′_predicted_ × PPFR) is the sum of the photochemical energy uptake of the intercepted leaf area between germination and senescence, and $\sum_{\mathrm{Senescence}}^{\mathrm{Germination}}$PPFR is the sum of intercepted sunlight energy in the photosynthetic active range between germination and senescence in µmol photons m^−2^. The *ɛ_i_* is cancelled from both sums, that is, *ɛ_e_* is not dependent on *ɛ_i_*. The *ɛ_e_* was calculated for every genotype (maize) or every plot (soybean) in every experiment. Note that when *ɛ_e_* is calculated with Response_G:PPFR_, it follows that:

(10) $$\varepsilon_{e}\propto {\text{Response}}_{G:\text{PPFR}}$$

since in that case ∑SenescenceGermination( F q'/F m ′predicted×PPFR) equals ∑SenescenceGermination (ResponseG:PPFR×PPFR)2.

#### Energy content of biomass

Energy content of dried biomass was assumed to be 18 MJ kg^−1^ in all samples (McKendry, 2002). Since no root biomass data were available, it was approximated as 9% and 17% of maize and soybean total biomass, respectively, as determined in Ordóñez et al. (2020). Since no stover biomass for the maize field data was available, it was approximated as 39% of the total biomass (Ordóñez et al., 2020). In that way, measured biomass per square meter was converted to total biomass per square meter (in J m^−2^). Conversion of PPFR to J s^−1^ m^−2^ was done by applying a factor of 0.219 µmol photons^−1^ (Langhans et al., 1997).

#### Transduction efficiency of photochemical energy into biomass

The *ɛ_t_* was derived according to Equation (2) dividing the derived total biomass per square meter by *ɛ_e_* calculated based on *F*_q_′/*F*_m_′ according to Equation (9), by $\sum_{\mathrm{Senescence}}^{\mathrm{Germination}}$PPFR and by *ɛ_i_* assumed as 0.9 as described above. The adjusted genotypic means of *ɛ_e_* respective ɛ_t_ were calculated using the different experiments as fixed effects.

### Data availability

The data sets generated and analyzed for this study are available in the zipped supplemental data file: The LIFT (Supplemental Data S1), the weather (Supplemental Data S2), the biomass (Supplemental Data S3), and the LI-COR (Supplemental Data S4) data set.

## Supplemental data  

The following materials are available in the online version of this article.

**Supplemental Figure S1.** Relative importance of environmental and spectral coefficients for photosynthetic quantum efficiency (*F*q′/*F*m′) in maize and soybean are shown.

**Supplemental Figure S2.** Photosynthetic quantum efficiency (*F*_q_′/*F*_m_′) of soybean and maize genotypes was modeled with PPFR and related to biomass.

**Supplemental Figure S3.** Photosynthesis in maize and soybean genotypes over time was correlated to their biomass in five different environments.

**Supplemental Figure S4.** Observed and imputed spectral variables for a subset of 7 d.

**Supplemental Figure S5**. Photosynthetic quantum efficiency (*F*_q_′/*F*_m_′) of soybean genotypes were predicted based on half of the measuring days (training set) and correlated with the data of the remaining days (validation set) in order to assess prediction accuracy.

**Supplemental Figure S6.** ET was estimated over entire growing seasons and correlated to biomass.

**Supplemental Figure S7.** Efficiency of photosynthetic ET 5 ms after primary quinone reduction (*F*_r2_′/*F*_q_′) of soybean and maize genotypes was related to temperature and biomass.

**Supplemental Table S1.** Description of all experiments with site, crop, genotype, treatment, year, sowing, and harvest data and the number of replicates (Rep).

**Supplemental Data S1.** Supplemental_Data_S1_LIFT_data.csv.

**Supplemental Data S2.** Supplemental_Data_S2_Weather_data.csv.

**Supplemental Data S3.** Supplemental_Data_S3_Biomass_data.csv.

**Supplemental Data S4.** Supplemental_Data_S4_LI-COR_data.csv.

## Supplementary Material

kiab483_Supplementary_Data

## Notes

O.M., B.K., S.M., and U.R. designed the study. B.K., L.Z., A.S., and O.M. set up the instruments and platforms, carried out the experiments and measurements. B.K. and L.Z. analyzed the data. B.K. wrote the manuscript with contributions from S.M., U.R., and O.M.

The author responsible for distribution of materials integral to the findings presented in this article in accordance with the policy described in the Instructions for Authors (https://academic.oup.com/plphys/pages/general-instructions) is Beat Keller (kellebea@alumni.ethz.ch).
