# Supplementary material for: Toward predicting photosynthetic efficiency and biomass gain in crop genotypes over a field season
Source: Plant Physiol. 2021 Oct 18;188(1):301–17. doi: 10.1093/plphys/kiab483 (PMC8774793; doi:10.1093/plphys/kiab483)
Supplement: kiab483_Supplementary_Data [file kiab483_supplementary_data.zip › kiab483-suppl_data/Kelleretal_Supplemental_Material.pdf]

Supplemental material

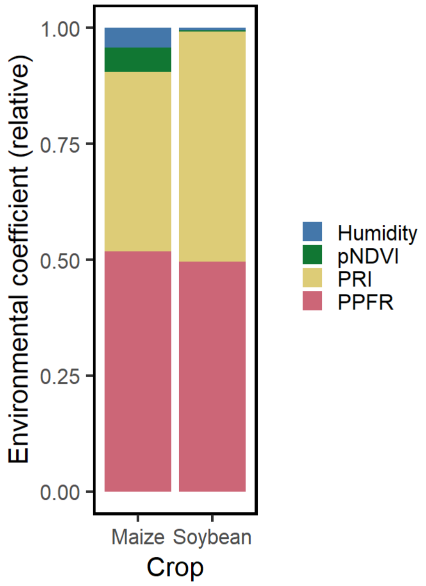

Supplemental Figure S1 Relative importance of environmental and spectral coefficients for photosynthetic quantum efficiency (F_q'/F_m') in maize and soybean are shown. Predictors, i.e., air humidity, pseudo normalized difference vegetation index (pNDVI), photochemical reflectance index (PRI) and photosynthetic photon fluence rate (PPFR), were standardized before calculating model (3) and (4).

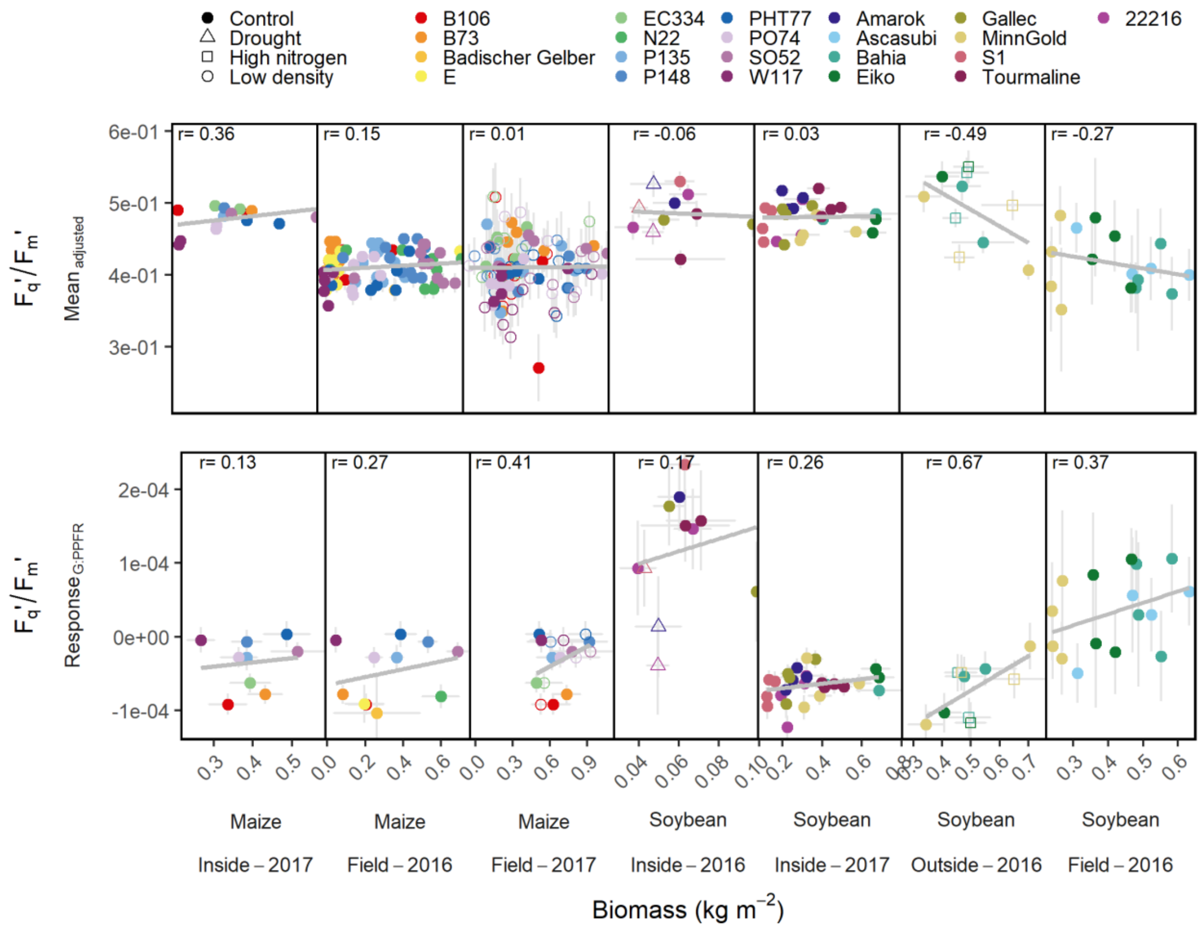

Supplemental Figure S2 Photosynthetic quantum efficiency (F_q'/F_m') of soybean and maize genotypes was modeled with photosynthetic photon fluence rate (PPFR) and related to biomass. The models (3) and (4) were used without spectral variables. The adjusted F_q'/F_m' means and the genotypic responses of F_q'/F_m' to PPFR ($Response_{G:PPFR}$) were correlated with biomass in all seven experiments. In soybean, the $Response_{G:PPFR}$ was calculated separately for each container or plot according to equation (5) using model (3), whereas in maize it was adjusted over all containers and plots using model (4) due to the lower amount of measurements. For this modeling, measurements were previously averaged for every minute, genotype and repetition. Grey error bars show the standard error of the adjusted mean respective response (n=8 to 504 for F_q'/F_m; n=1 to 11 for biomass). Plants were grown under control conditions, except three containers were subjected to drought, five containers were fertilized with additional nitrogen (high N) and eight maize genotypes were planted in low density in 2017.

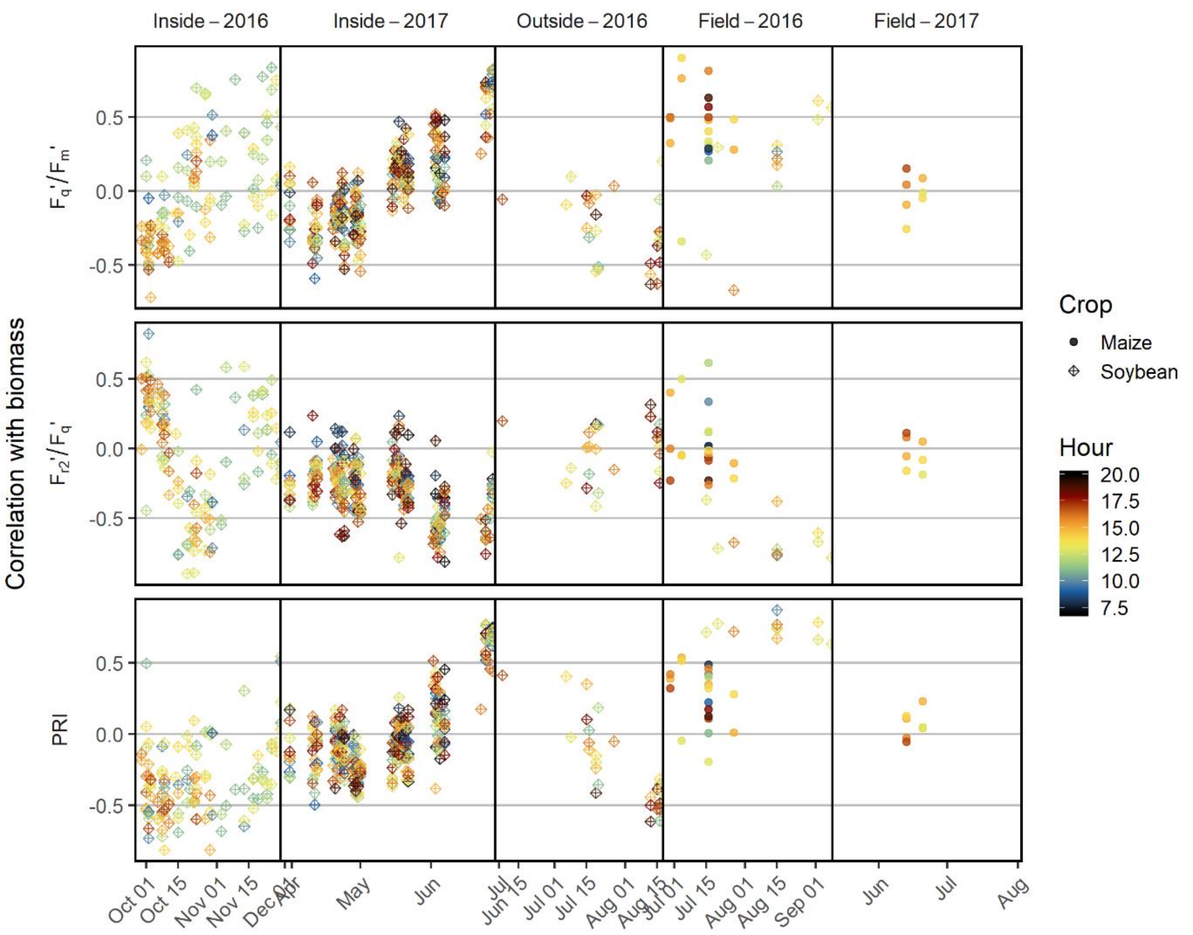

Supplemental Figure S3 Photosynthesis in maize and soybean genotypes over time was correlated to their biomass in five different environments. Automated light-induced fluorescence transient (LIFT) systems scanned plant canopies inside and outside of the glasshouse as well as in the field. Operating efficiency of photosystem II (F_q'/F_m'), efficiency of photosynthetic electron transport 5 ms after primary quinone reduction (F_{r2}'/F_q'), and photochemical reflectance index (PRI) were correlated with accumulated biomass at every hour of available measurement data.

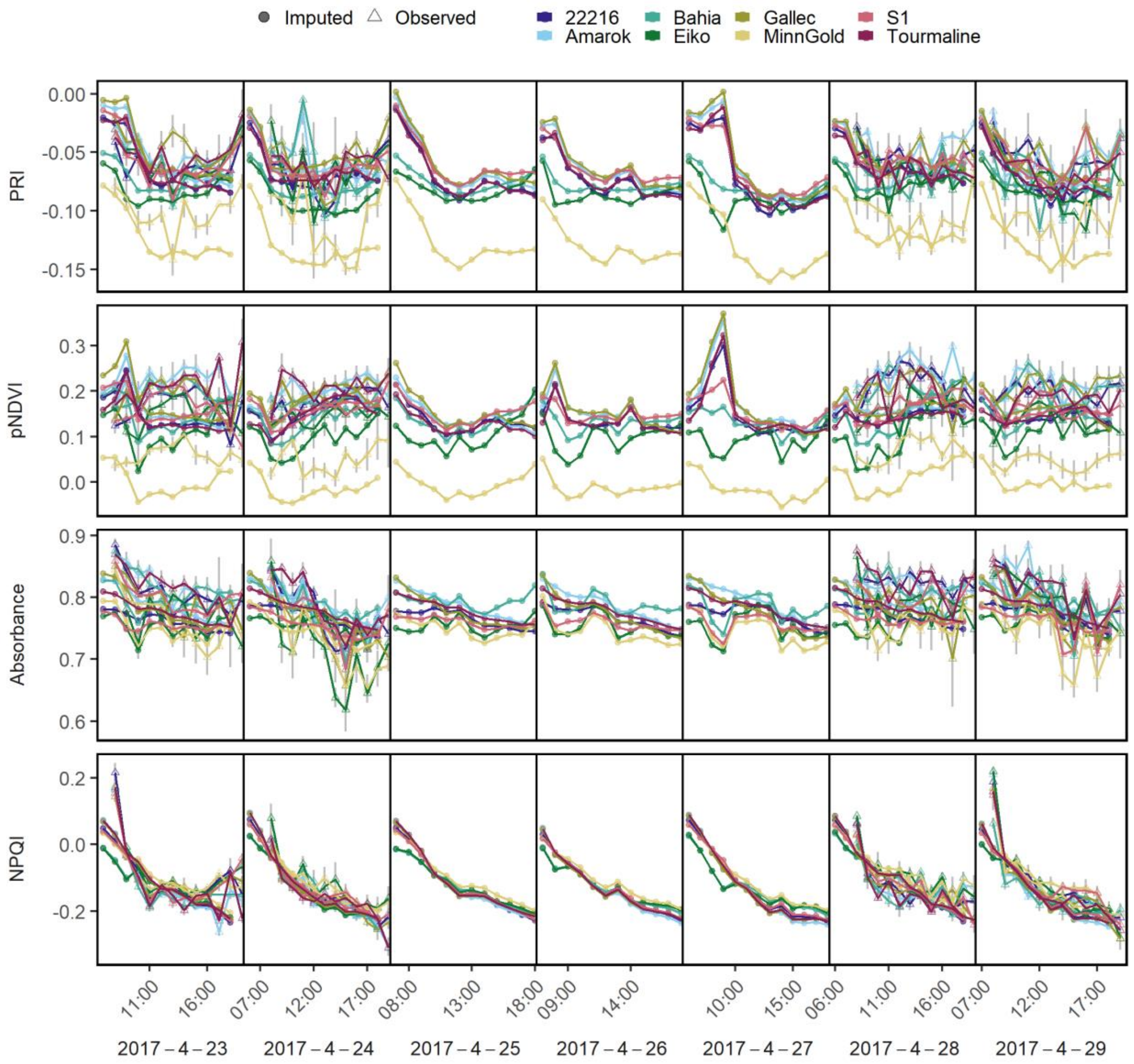

Supplemental Figure S4 Observed and imputed spectral variables for a subset of seven days. Missing data of photochemical reflectance index (PRI), pseudo normalized difference vegetation index (pNDVI), absorbance and normalized phaeophytinization index (NPQI) were imputed based on measured environmental and spectral data. Grey error bars show standard error of the mean per hour and genotype (n=1 to 8, total measured n=2104).

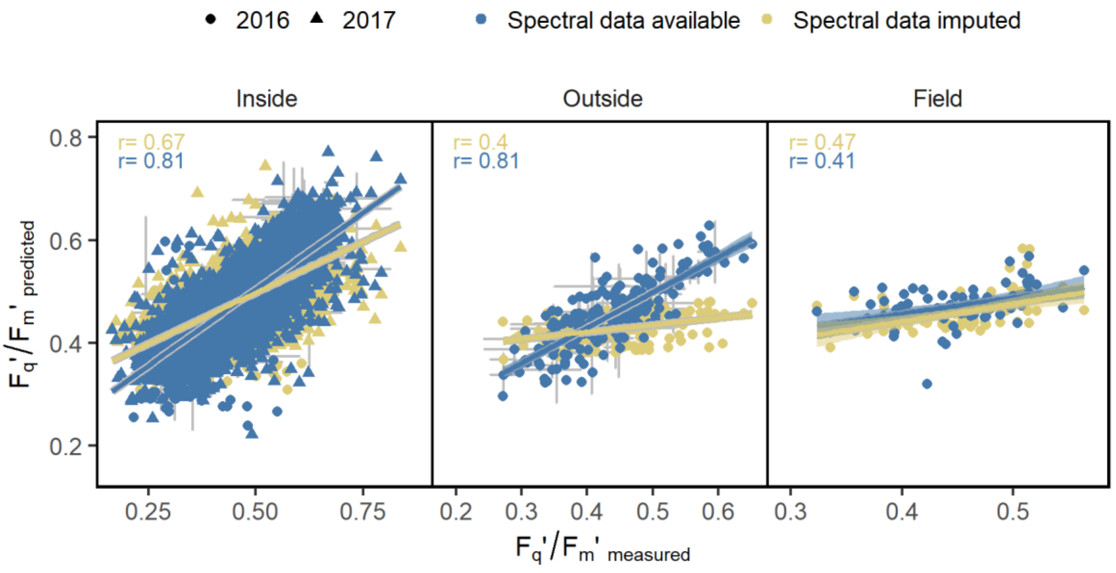

Supplemental Figure S5 Photosynthetic quantum efficiency (F_q'/F_m') of soybean genotypes were predicted based on half of the measuring days (training set) and correlated with the data of the remaining days (validation set) in order to assess prediction accuracy. This procedure was done once with available spectral data in the validation set and once when the spectral data had to be imputed first. The prediction accuracy is shown for the three growth environments: containers inside and outside of the glasshouse and plots in the field. Predicted and measured values were averaged per hour and genotypes (n=1 to 8, total measured n=20,721). Grey error bars show standard deviation of the mean.

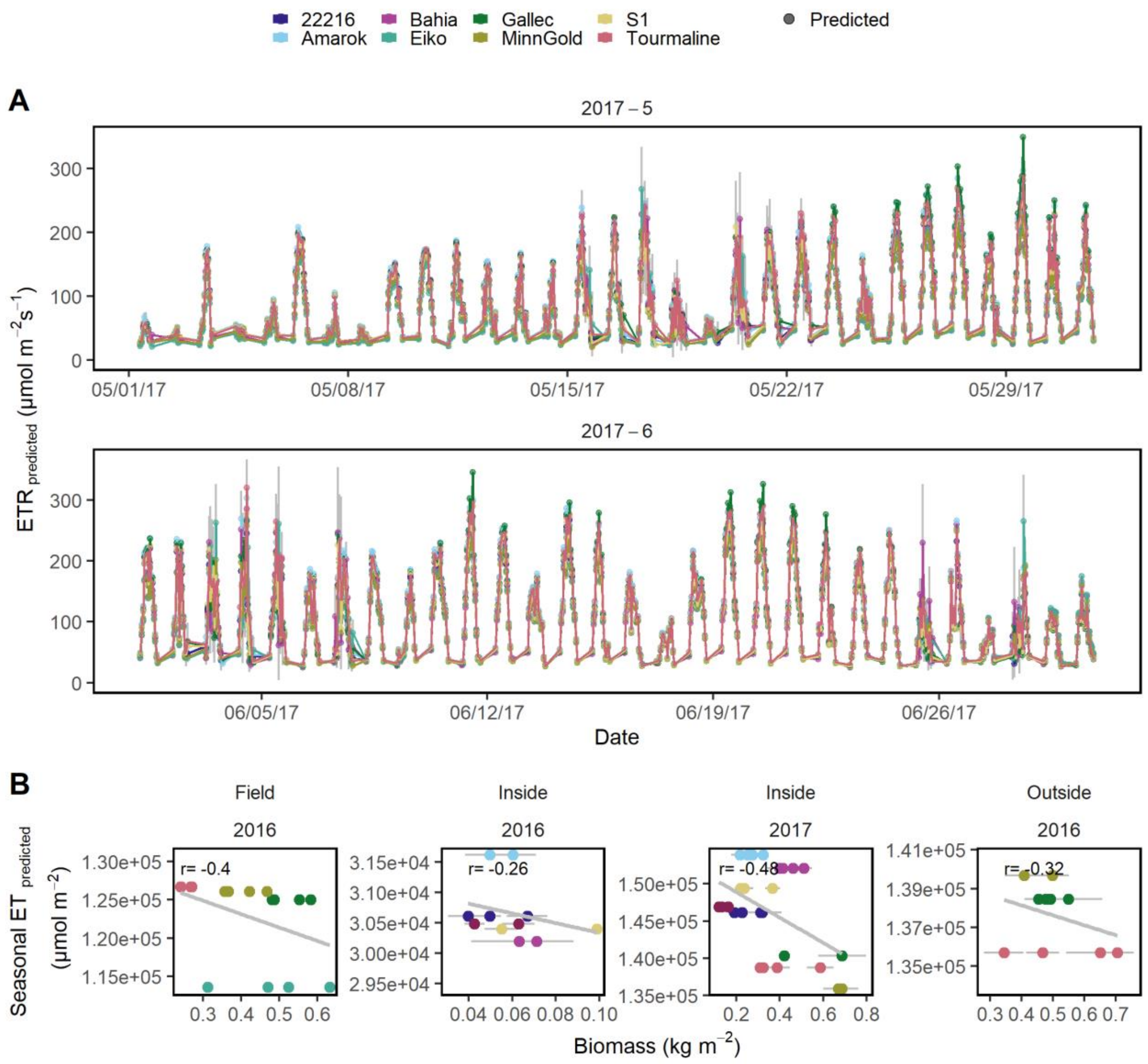

Supplemental Figure S6 Electron transport (ET) was estimated over entire growing seasons and correlated to biomass. A: Electron transport rates (ETR) were calculated for every hour of the full growing season. Grey lines show residual error as estimated minus measured value. B: Correlations between cumulated seasonal ET and biomass production are shown. Grey error bars show the standard error of the mean for each plot or container (n=1 to 11 for biomass).

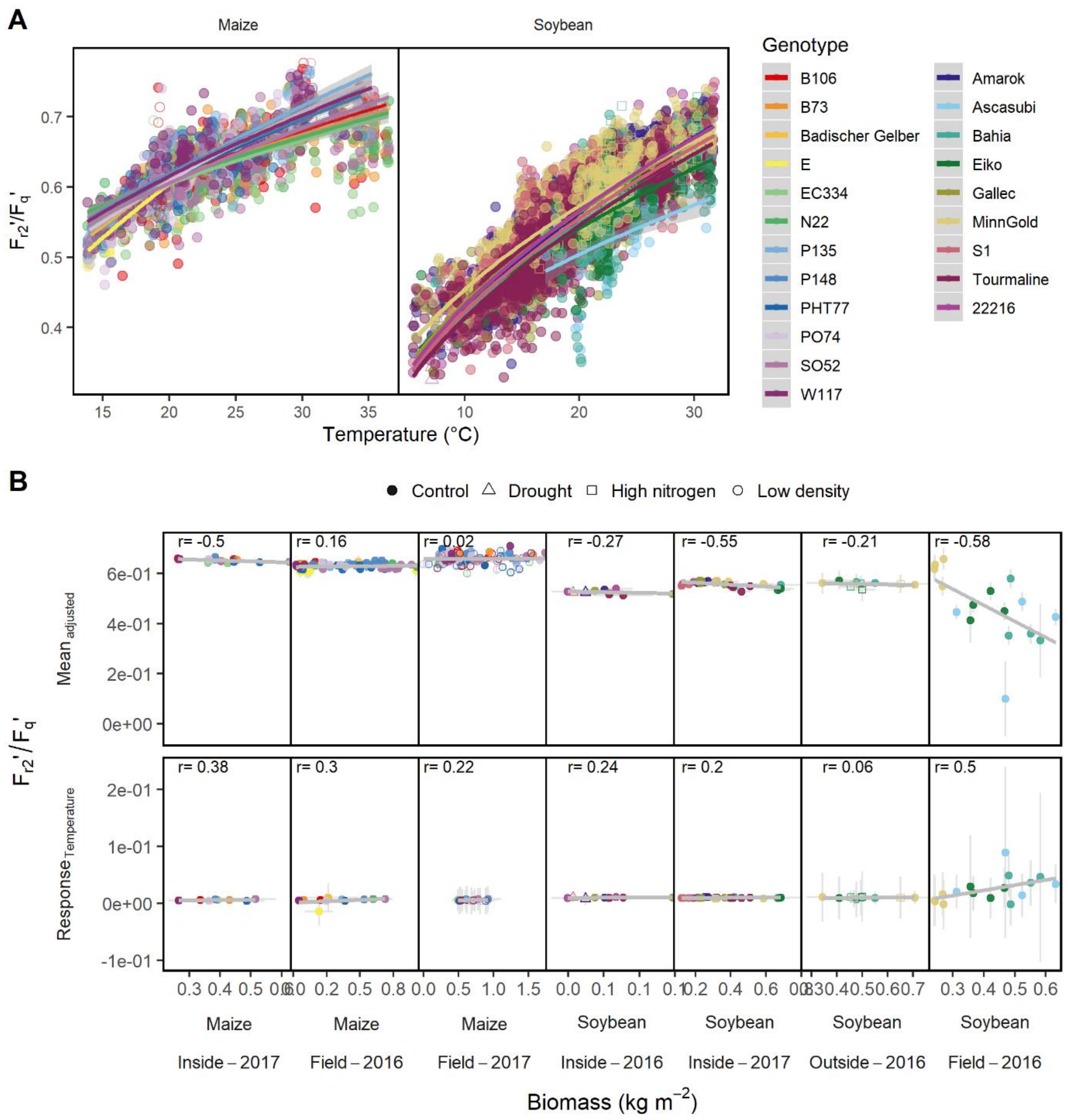

Supplemental Figure S7 Efficiency of photosynthetic electron transport 5 ms after primary quinone reduction (F_{r2}'/F_q') of soybean and maize genotypes was related to temperature and biomass. A: The response of adjusted F_{r2}'/F_q' to temperature is shown for every genotype. B: The adjusted F_{r2}'/F_q' means and the genotypic responses of F_{r2}'/F_q' to temperature were correlated with biomass in all seven experiments. Grey error bars show the standard error of the estimated mean respective response. In soybean the response to temperature was calculated separately for each container or plot whereas in maize it was adjusted over all containers and plots due to the lower amount of measurements. For this modeling, measurements were previously averaged for every minute, genotype and repetition. Grey error bars show the standard error of the adjusted mean respective response (n=8 to 504 for F_{r2}'/F_q'; n=1 to 11 for biomass). Plants were grown under control conditions, except three containers were subjected to drought, five containers were fertilized with additional nitrogen (high N) and eight maize genotypes were planted in low density in 2017. The model (3) and (4) were adapted for F_{r2}'/F_q' with days after sowing instead of photochemical reflectance index (PRI) since PRI has no major influence on F_{r2}'/F_q'.

Supplemental Table S1 Description of all experiments with site, crop, genotype, treatment, year, sowing and harvest data and the number of replicates (Rep).

Site	Crop	Genotype	Treatment	Year	Sowed_Date	Harvest_Date	Rep
Field	Maize	SO52	Control	2017	08.05.2017	24.10.2017	10
Field	Maize	B73	Control	2017	08.05.2017	24.10.2017	10
Field	Maize	P135	Control	2017	08.05.2017	24.10.2017	10
Field	Maize	PO74	Low density	2017	08.05.2017	24.10.2017	8
Field	Maize	EC334	Low density	2017	08.05.2017	24.10.2017	8
Field	Maize	W117	Control	2017	08.05.2017	24.10.2017	10
Field	Maize	B106	Control	2017	08.05.2017	24.10.2017	10
Field	Maize	EC334	Control	2017	08.05.2017	24.10.2017	10
Field	Maize	PO74	Control	2017	08.05.2017	24.10.2017	10
Field	Maize	P148	Low density	2017	08.05.2017	24.10.2017	8
Field	Maize	W117	Low density	2017	08.05.2017	24.10.2017	7
Field	Maize	PHT77	Control	2017	08.05.2017	24.10.2017	10
Field	Maize	SO52	Low density	2017	08.05.2017	24.10.2017	8
Field	Maize	PHT77	Low density	2017	08.05.2017	24.10.2017	8
Field	Maize	P148	Control	2017	08.05.2017	24.10.2017	10
Field	Maize	B106	Low density	2017	08.05.2017	24.10.2017	8
Field	Maize	PO74	Control	2016	06.05.2016	08.11.2016	7
Field	Maize	N22	Control	2016	06.05.2016	08.11.2016	10
Field	Maize	P135	Control	2016	06.05.2016	08.11.2016	10
Field	Maize	P148	Control	2016	06.05.2016	08.11.2016	10
Field	Maize	B73	Control	2016	06.05.2016	08.11.2016	10
Field	Maize	SO52	Control	2016	06.05.2016	08.11.2016	10
Field	Maize	E	Control	2016	06.05.2016	08.11.2016	10
Field	Maize	Badischer Gelber	Control	2016	06.05.2016	08.11.2016	11
Field	Maize	PHT77	Control	2016	06.05.2016	08.11.2016	8
Field	Maize	W117	Control	2016	06.05.2016	08.11.2016	10
Field	Maize	B106	Control	2016	06.05.2016	08.11.2016	4
Field	Soybean	Eiko	Field	2016	01.07.2016	18.10.2016	4
Field	Soybean	Ascasubi	Field	2016	01.07.2016	18.10.2016	4
Field	Soybean	MinnGold	Field	2016	01.07.2016	25.10.2016	4
Field	Soybean	Bahia	Field	2016	01.07.2016	11.10.2016	4
Inside	Maize	SO52	Control	2016	24.05.2016	07.07.2016	2
Inside	Maize	N22	Control	2016	24.05.2016	07.07.2016	2
Inside	Maize	EC334	Control	2016	24.05.2016	07.07.2016	2
Inside	Maize	B106	Control	2016	24.05.2016	07.07.2016	2
Inside	Maize	B73	Control	2016	24.05.2016	07.07.2016	2

Inside	Maize	PO74	Control	2017	30.05.2017	14.08.2017	3
Inside	Maize	P148	Control	2017	30.05.2017	14.08.2017	2
Inside	Maize	EC334	Control	2017	30.05.2017	14.08.2017	2
Inside	Maize	B106	Control	2017	30.05.2017	14.08.2017	2
Inside	Maize	PHT77	Control	2017	30.05.2017	14.08.2017	2
Inside	Maize	P135	Control	2017	30.05.2017	14.08.2017	1
Inside	Maize	B73	Control	2017	30.05.2017	14.08.2017	2
Inside	Maize	SO52	Control	2017	30.05.2017	14.08.2017	2
Inside	Maize	W117	Control	2017	30.05.2017	14.08.2017	2
Inside	Soybean	Bahia	Control	2017	08.03.2017	07.08.2017	2
Inside	Soybean	Tourmaline	Control	2017	08.03.2017	07.08.2017	4
Inside	Soybean	Amarok	Control	2017	08.03.2017	07.08.2017	4
Inside	Soybean	22216	Control	2017	08.03.2017	07.08.2017	4
Inside	Soybean	MinnGold	Control	2017	08.03.2017	07.08.2017	4
Inside	Soybean	Eiko	Control	2017	08.03.2017	07.08.2017	2
Inside	Soybean	Gallec	Control	2017	08.03.2017	07.08.2017	4
Inside	Soybean	S1	Control	2017	08.03.2017	07.08.2017	4
Inside	Soybean	22216	Drought	2016	19.08.2016	05.12.2016	2
Inside	Soybean	22216	Control	2016	19.08.2016	05.12.2016	2
Inside	Soybean	S1	Control	2016	19.08.2016	05.12.2016	1
Inside	Soybean	Tourmaline	Control	2016	19.08.2016	05.12.2016	2
Inside	Soybean	Amarok	Control	2016	19.08.2016	05.12.2016	2
Inside	Soybean	Amarok	Drought	2016	19.08.2016	05.12.2016	1
Inside	Soybean	S1	Drought	2016	19.08.2016	05.12.2016	1
Inside	Soybean	Gallec	Control	2016	19.08.2016	05.12.2016	2
Outside	Soybean	MinnGold	Control_Outside	2016	08.05.2016	24.09.2016	2
Outside	Soybean	Eiko	Control_Outside	2016	08.05.2016	24.09.2016	1
Outside	Soybean	Eiko	High nitrogen	2016	08.05.2016	24.09.2016	1
Outside	Soybean	Bahia	High nitrogen	2016	08.05.2016	24.09.2016	2
Outside	Soybean	Bahia	Control_Outside	2016	08.05.2016	24.09.2016	2
Outside	Soybean	MinnGold	High nitrogen	2016	08.05.2016	24.09.2016	2
